# Mindfulness- and acceptance-based interventions for patients with fibromyalgia – A systematic review and meta-analyses

**DOI:** 10.1371/journal.pone.0221897

**Published:** 2019-09-03

**Authors:** Trond Haugmark, Kåre Birger Hagen, Geir Smedslund, Heidi A. Zangi

**Affiliations:** 1 Department of Rheumatology, National Advisory Unit on Rehabilitation in Rheumatology, Diakonhjemmet Hospital, Oslo, Norway; 2 Faculty of Medicine, Institute of Health and Society, University of Oslo, Oslo, Norway; 3 Division of Health Service, Norwegian Institute of Public health, Oslo, Norway; 4 Faculty of Health, VID Specialized University, Oslo, Norway; University of Sao Paulo Medical School, BRAZIL

## Abstract

**Objectives:**

To analyze health effects of mindfulness- and acceptance-based interventions, including mindfulness-based stress reduction (MBSR), mindfulness-based cognitive therapy (MBCT) and acceptance and commitment therapy (ACT). Additionally, we aimed to explore content and delivery components in terms of procedure, instructors, mode, length, fidelity and adherence in the included interventions.

**Methods:**

We performed a systematic literature search in the databases MEDLINE, PsychINFO, CINAHL, EMBASE, Cochrane Central and AMED from 1990 to January 2019. We included randomized and quasi-randomized controlled trials analyzing health effects of mindfulness- and acceptance-based interventions for patients with fibromyalgia compared to no intervention, wait-list control, treatment as usual, or active interventions. MBSR combined with other treatments were included. Predefined outcomes were pain, fatigue, sleep quality, psychological distress, depression, anxiety, mindfulness, health-related quality of life and work ability. The Template for Intervention Description and Replication (TIDieR) checklist and guide was used to explore content and delivery components in the interventions. Meta-analyses were performed, and GRADE was used to assess the certainty in the evidence.

**Results:**

The search identified 4430 records, of which nine original trials were included. The vast majority of the participants were women. The analyses showed small to moderate effects in favor of mindfulness- and acceptance-based interventions compared to controls in pain (SMD -0.46 [95% CI -0.75, -0.17]), depression (SMD -0.49 [95% CI -0.85, -0.12]), anxiety (SMD -0.37 [95% CI -0.71, -0.02]), mindfulness (SMD -0.40 [-0.69, -0.11]), sleep quality (SMD -0.33 [-0.70, 0.04]) and health-related quality of life (SMD -0.74 [95% CI -2.02, 0.54]) at end of treatment. The effects are uncertain due to individual study limitations, inconsistent results and imprecision.

**Conclusion:**

Health effects of mindfulness- and acceptance-based interventions for patients with fibromyalgia are promising but uncertain. Future trials should consider investigating whether strategies to improve adherence and fidelity of mindfulness- and acceptance-based interventions can improve health outcomes.

## Introduction

Fibromyalgia (FM) is a complex and heterogeneous condition that may have important impact on patients’ quality of life. Pain is the dominant symptom, but other symptoms such as non-refreshed sleep, fatigue, mood disturbance and cognitive impairment are common [1]. Current pharmacological treatments for FM are non-curative. European League Against Rheumatism (EULAR) recommends non-pharmacological therapies as first-line therapy. Individualized physical exercise should be recommended for all patients with FM. Additionally, cognitive behavioral therapy, mindfulness-based stress reduction, meditative movement, hydrotherapy or a combination of these therapies have shown promising effects for some patients, but the evidence is still insufficient [1].

Physical and emotional stress-provoking life events and stress resulting from living with pain may play a role in development of FM and exacerbation of the symptoms [2, 3]. Hence, techniques that can help patients cope with their stress-related experiences may reduce symptoms and improve wellbeing [4]. Mindfulness- and acceptance-based interventions, such as mindfulness-based stress reduction (MBSR) [5], mindfulness-based cognitive therapy (MBCT) [6] and acceptance and commitment therapy (ACT) [7] address peoples´ relationship to their internal experiences. The interventions aim to train the participants to intentionally observe thoughts, emotions, and bodily sensations as they are perceived on a moment-to-moment basis with an open, non-judgmental attitude [8]. It is argued that when experiences are not judged as good or bad, positive or negative, acceptance increases and one’s struggle to control what might not be controllable decreases. This should eventually lead to greater self-care and self-compassion [9].

MBSR and MBCT are typically provided as eight weekly 2–2.5 hours’ group sessions plus a one-day retreat. They include practical and formal meditation training, such as body scan, sitting and walking meditation, mindful yoga movements and individual practice between sessions [5]. MBCT was adapted from MBSR to prevent recurrent depressions and replaces some of the content of MBSR with education on specific patterns of negative thinking that people with depression are vulnerable to [6]. ACT consists of a set of treatment methods and aims to focus on other cognitive skills such as the participants’ ability to define and clarify values in different life domains, identify achievable goals that embody those values, and plan the future based on identified life goals. ACT can be delivered in a wide array of applications including group treatment, individual treatment, via internet and self-administered workbooks with therapist support [10]. ACT is delivered to a variety of patient populations and settings and focuses on different problems, e.g. depression, anxiety, chronic pain or the management of chronic disease [11–13]. Ultimately, the purpose is to develop psychological flexibility, which refers to the capacity to change or maintain one’s behavior in open contact with thoughts and feelings [12, 14].

Systematic reviews and meta-analyses on mindfulness- and acceptance-based interventions for chronic pain conditions have showed a number of beneficial effects in patients with chronic pain, especially in the long-term [15, 16]. For patients diagnosed with FM, the evidence is scarcer. A recent systematic review and meta-analysis showed that cognitive behavioral therapies (CBT), including CBT, MBCT and ACT reduced key symptoms such as pain, negative mood and disability compared to control interventions [17]. Another systematic review on MBSR for patients with FM showed that MBSR improved health-related quality of life and reduced pain intensity compared to usual care and active control groups [18].

There has been an upsurge of mindfulness- and acceptance-based interventions across different fields of research. Although the interventions are relatively well described, there may be differences in how they are delivered and implemented [19], and details of the interventions may be lacking [20]. Hence, researchers may find it difficult to replicate the interventions in future trials [21]. Moreover, for clinicians it may be challenging to implement the interventions in clinical practice [22]. In the present systematic review we have applied the Template for Intervention Description and Replication (TIDieR) checklist and guide [23] (S1 Table) to explore the intervention components in MBSR, MBCT and ACT as they were presented in randomized controlled trials.

The aims of this study were:

- to explore content and delivery components in mindfulness- and acceptance-based interventions for patients with FM
- to analyze health effects of mindfulness- and acceptance-based interventions for patients with FM

## Materials and method

This systematic review was performed according to the Cochrane Handbook [24] and reported according to the Preferred Reporting Items of Systematic Reviews and Meta-Analyses (PRISMA) [25]. The reviewers comprised two experts on mindfulness- and acceptance-based interventions (TH, HAZ) and two methodologists (GS, KBH). Inclusion criteria, methods and analyses were specified in advance, and the protocol was registered in PROSPERO (CRD42018081119).

### Search methods for identification of studies

The electronic databases MEDLINE, PsychINFO, CINAHL, EMBASE, Cochrane Central and AMED were searched from 1990 to January 25^th^ 2019. A medical librarian developed the MEDLINE search strategy in consultation with the reviewers. The strategy was amended for each database (S1 Text) and restricted to English, Swedish, Danish, Norwegian, German, French, Spanish and Portuguese languages. The reference lists of included studies were examined for additional potentially eligible studies.

### Eligibility criteria

We included randomized controlled trials (RCTs) and quasi-randomized trials on mindfulness- and acceptance-based interventions for patients with FM. Only full-text articles published in peer-reviewed journals were included. The study population was limited to adult patients (age ≥ 18) diagnosed with FM based on the initial criteria defined by the American College of Rheumatology (ACR) in 1990 [26] or the revised ACR 2010 criteria [27].

Studies were considered if they followed the standardized format of MBSR, MBCT or ACT and were compared to no intervention, wait-list control, treatment as usual, or active interventions. Modified interventions were considered for inclusion if they referred to the originators of the interventions in the reference list; for MBSR: Kabat-Zinn, MBCT: Teasdale, Segal, Williams, and ACT: Hayes. The interventions had to comprise 6 to 12 sessions and include group-based or online mindfulness meditation over at least 6 weeks.

Studies were included if they assessed at least one of the outcomes pain, fatigue, sleep quality, psychological distress, depression, anxiety, mindfulness, health-related quality of life or work ability. Effects were categorized as end-of-treatment and follow-up scores (2 to 6 months).

### Selection of studies

After duplicate removal, two reviewers (TH and GS) independently screened the titles and abstracts and selected studies based on the inclusion and exclusion criteria, using Rayyan screening tool [28]. The articles selected for full-text screening were examined independently by the same reviewers. Disagreements were rechecked and consensus was achieved by discussion before the final selection. A third reviewer (HAZ) was consulted in cases of dissension. When needed, study authors were contacted for additional information to clarify study eligibility and obtain further details.

### Data extraction and management

General information, population, setting, methods, and participant data were extracted by one reviewer (TH) and checked by a second reviewer (GS) using a data extraction form created for the review (S2 Table).

TH and GS used the TIDieR-checklist to extract the intervention components in each study (S1 Table). Disagreements were rechecked and consensus achieved by discussion before the final checklist was completed.

### Assessment of methodological quality

The Cochrane risk of bias tool [24] was used with six domains separately assessed: sequence generation, allocation concealment, blinding of participants, personnel and outcome assessors, incomplete outcome data, selective reporting and other potential threats to validity. Each domain was explicitly rated by two reviewers (TH and GS) as low, high or unclear risk of bias (S1 Fig).

GRADEpro [29] was used to rate and summarize the certainty of evidence of the reported outcomes as high, moderate, low, and very low. As only RCTs were included, the rating started at high certainty and was downgraded by one or two levels for concerns in one of the five domains; study limitations, inconsistent results, indirectness of evidence, imprecision and publication bias [30].

### Statistical analysis

If the studies were sufficiently similar regarding participants, interventions, comparisons and outcomes, we conducted meta-analyses using Review Manager software (RevMan 5.3) from the Cochrane collaboration [31]. Due to clinical heterogeneity, we decided to perform and report random-effects analyses. Heterogeneity was assessed using tau-squared and I-squared statistics [32]. Considering shortcomings of the tau-squared estimator in RevMan (Der Simonian and Laird [DL]) we repeated the analyses with the Hartung-Knapp-Sidik-Jonkman (SJ) estimator in R Studio Version 1.1.463 to verify the results [33]. Since the SJ results did not differ substantially from the DL results, we decided to keep the results from the RevMan analyses. We computed standardized mean differences (SMDs) because different scales were used to measure the same outcomes.

Controls included were no intervention, wait-list controls, treatment as usual and other active interventions. In studies with more than one control arm, we calculated weighted averages. The mindfulness and health-related quality of life scales were inverted to be comparable to the other outcomes.

## Results

### Selection process

**Fig 1:**
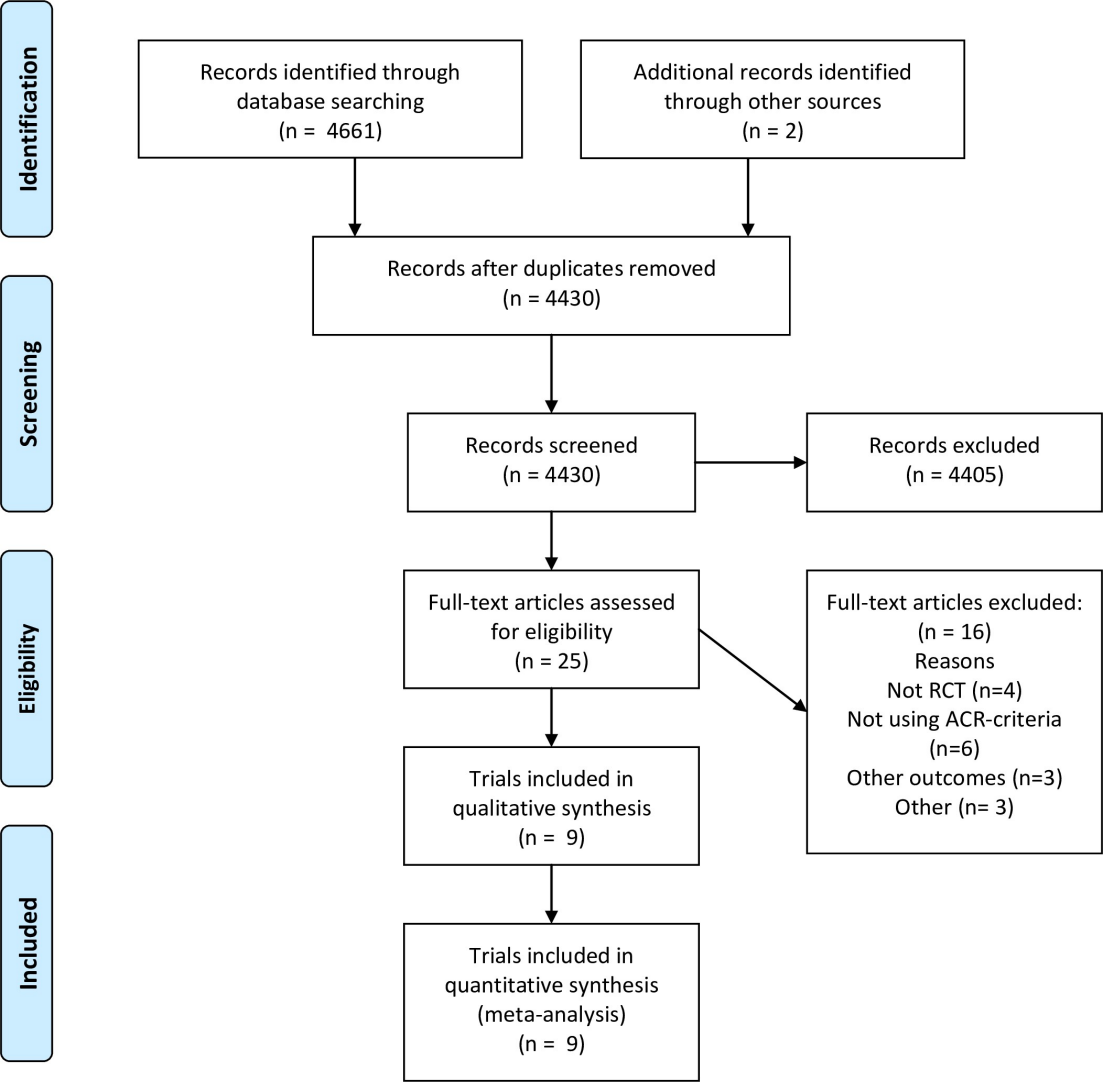
PRISMA flow diagram. PRISMA Study flow diagram.

Twenty-five of 4430 articles were identified as potentially eligible and screened in full-text (Fig 1). Nine trials met the inclusion criteria. The reasons for exclusion of 16 trials are presented in S3 Table.

### Trial characteristics

The included trials were published between 2003 and 2018 totaling 750 participants (Table 1).

**Table 1:** Characteristics of included studies (n = 9).

Author, year, country (Ref)	Study design	Participants	Intervention	Comparison (control)	Outcome measures	Measurement time point
**Astin et al. 2003, USA [34]**	RCT	128 participants, mean age 48 yrs., 98.4% women in intervention and 100% in control group	n = 64, mindfulness meditation/ qigong movement therapy	n = 64, education-support group	Pain; MOS SF-36, BDI	Baseline, end of treatment (8 weeks), and at follow-up (4 months + 6 months)
**Cash et al. 2015, USA [41]**	RCT	91 participants, mean age 48 yrs., 100% women	n = 51, mindfulness-based stress reduction	n = 40, wait-list	Pain; VAS, SSQ, The Fatigue Symptom Inventory	Baseline, end of treatment (8 weeks) and at follow-up (2 months)
**Grossman et al. 2007, Switzerland [35]**	Quasi-RCT	58 participants, mean age 52 yrs., 100% women	n = 38, mindfulness-based stress reduction	n = 13, education-support group	Pain; VAS, HADS, QoL*	Baseline and at end of treatment (8 weeks)
**Luciano et al. 2014, Spain [36]**	RCT	156 participants, mean age 48 yrs., 96% women in both groups	n = 51, acceptance and commitment therapy	n = 52, recommended pharmacological treatment + n = 53, wait-list	HADS, Pain; VAS, EQ-5D	Baseline, end of treatment (8 weeks) and follow-up (6 months)
**Parra-Delgado et al. 2013, Spain [37]**	RCT	33 participants, mean age 53 yrs., 100% women	n = 17, mindfulness-based cognitive therapy	n = 16, treatment as usual	BDI, Pain; VAS**	Baseline, end of treatment (8 weeks) and follow-up (3 months)
**Schmidt et al. 2011, Germany [38]**	RCT	177 participants, mean age 53 yrs., 100% women	n = 59, mindfulness-based stress reduction	n = 59, education-support group + n = 59, wait-list	HRQoL (PLC), CES-D, STAI, PSQI, PPS***, FMI	Baseline, end of treatment (8 weeks) and follow-up (2 months)
**Septhon et al. 2007, USA [39]**	RCT	91 participants, mean age 48 yrs., 100% women	n = 51, mindfulness-based stress reduction	n = 40, wait-list	BDI	Baseline, end of treatment (8 weeks) and at follow-up (2 months)
**Simister et al. 2018, Canada [42]**	RCT	67 participants, mean age 40 yrs., 95% women	n = 33, acceptance and commitment therapy	n = 34, treatment as usual	CES-D, SF-MPQ, PSQI, FFMQ	Baseline, end of treatment (12-weeks) and follow-up (3 months)
**Wicksell et al. 2012, Sweden [40]**	RCT	40 participants, mean age 45 yrs., 100% women	n = 23, acceptance and commitment therapy	n = 17, wait-list	PDI, SF-36****, BDI, STAI	Baseline, end of treatment (12-weeks) and follow-up (3–4 months)

RCT = Randomized Controlled Trial

BDI = Beck Depression Inventory; 21-question multiple-choice self-report inventory. Each question had a set of at least four possible responses, ranging in intensity

MOS SF-36 = Medical Outcome Study Shortform-36 Scores range from 0–100, Lower scores = more disability, higher scores = less disability

VAS = Visual Analogue Scale for pain intensity, 0–100, “no pain” (score of 0) and “pain as bad as it could be” or “worst imaginable pain” (score of 100)

SSQ = Stanford Sleep Questionnaire; 7-point scale with scale rating from 1 "feeling active, vital, alert, or awake" to 7 "No longer fighting sleep, sleep onset soon; having dream-like thoughts"

The Fatigue Symptom Inventory = scale composed of 14 items (one of which is not scored) designed to evaluate multiple aspects of fatigue, including its perceived severity, frequency, and interference with daily functioning

HRQoL (PLC) = The Quality of life Profile for the Chronically Ill; Questionnaire composed of 40 Likert-scaled items (scale 0–4) with 0 representing minimum and 4 representing maximum satisfaction. The items measure physical, psychological and social capacity of performance and well-being

HADS = Hospital Anxiety and Depression Scale; fourteen item scale that generates ordinal data. Seven of the items relate to anxiety and seven relate to depression

PPS = The Pain Perception Scale; 24-item scale that evaluates pain perception

EQ-5D = Visual analogue scale of EuroQol; EQ-5D self-reported questionnaire includes a visual analog scale (VAS), which records the respondent's self-rated health status on a graduated (0–100) scale, with higher scores for higher HRQoL. It also includes the EQ-5D descriptive system, which comprises 5 dimensions of health

CES-D = Center for Epidemiological Studies depression inventory; 20-item, self-report measure designed to measure symptoms of depression over the past week

STAI = State-Trait-Anxiety-Inventory; 20 items for assessing trait anxiety and 20 for state anxiety. All items are rated on a 4-point scale from 'not at all' to 'very much so'. Higher scores indicate greater anxiety

PSQI = Pittsburgh Sleep Quality Index; 19 individual items, creating 7 components producing one global score

FMI = Freiburg Mindfulness Inventory; a 14-item short form measuring Mindfulness

PDI = Pain Disability Index; a self-report tool used for measuring the degree of pain a patient is experiencing. Participants use a 0 (no disability) to 10 (total disability) numeric rating scale

PIPS = Psychological Inflexibility in Pain Scale; 16-item scale used to assess psychological inflexibility. Respondents are asked to rate items on a 7-point scale ranging from 1 (never true) to 7 (always true). Higher scores indicate greater levels of psychological inflexibility

SF-MPQ = Short form McGill Pain Questionnaire; 15 items asking participants to rank their typical pain experience on a 4-point Likert scale, from 0 (no pain) to 3 (severe), and maximum total score of 45

FFMQ = Five Facet Mindfulness Questionnaire; 39-items questionnaire measuring 5 facets of mindfulness

SF-36 = Short form-36 Healthy Survey, 36-item measure assessing health-related quality of life. Higher scores indicate better functioning

*The Quality of life Profile for the Chronically Ill was reported as six dimensions and therefore not included in this review

**The Visual Analogue Scale for pain intensity was measured on seven different parts of the body and not included in this review

***The Pain Perception Scale was divided into affective and sensory and only sensory was included in this review

****The short form-36 Healthy Survey provided summary for two subscales and not included in this review.

FM was diagnosed with ACR 1990 classification criteria in seven trials [34–40], and with ACR 2010 criteria in two trials [41, 42]. Two trials [38, 41] analyzed different outcomes within the same study population. Both were therefore included in the meta-analysis with the number of participants counted only once. Parra-Delgado et al. [37] measured pain in seven different areas of the body without reporting a sum score. The trial was therefore not included for pain outcomes in the meta-analysis. Simister et al. [42] delivered an internet-based ACT-intervention. Five authors were contacted to clarify diagnostic criteria and randomization procedures. Two authors provided the requested information; three did not respond.

### Exploration of content and delivery components in the interventions

The specific aims, content and mode of delivery of each trial are described in the TIDieR-checklist [23] (Table 2). The checklist revealed minor distinctions between trials in reporting and assessment of adherence and fidelity. These are reported below.

**Table 2:** TIDieR-checklist, template for intervention description and replication. Description of content and delivery components.

Author	Item 1+2, Brief name and Why	Item 3+4, What (materials and procedures)	Item 5, Who provided	Item 6, How	Item 7, Where	Item 8, When and How much	Item 9 + 10, Tailoring and Modification	Item 11, Strategies to improve or maintain intervention fidelity and adherence	Item 12, Extent of intervention fidelity and adherence
Astin et al. 2003 [34]	Mindfulness Meditation Plus Qigong Movement Therapy. Aim: to test the potential effect of Mindfulness and Qigong	First 90 minutes of each session based on MBSR, followed by 60 minutes introduction to qigong	Mindfulness instructors not reported. Qigong taught by Chinese master	Group—based (n = 10–20)	University	8 weeks, 8 2.5-hours, All-day retreat not reported	Not reported	Not reported	26% never attended a class. Of 128 randomized into 2 groups, 50 (39%) dropped out from the study prior to 'end of treatment', 61 (48%) dropped out by week 16, and 63 (49%) failed to complete 24 week assessment
Cash et al. 2015 [41]	MBSR alleviates FM symptoms in women. Aim: to test MBSR on physiological effects	MBSR (5). Home practice assignments	Trained MBSR instructors	Group-based (n = 10–12)	University	8 weeks, 8 2.5-hours, All-day retreat reported	Not reported	Attendance monitored and absent participants received a reminder phone call to attend subsequent sessions	Of 51 randomized to intervention 42 (82%) completed 5.5 sessions. Attendance rate dropped from 90% to 57% by 4th meeting and maintained between 57 and 65%. 68% of controls provided follow-up data
Grossman et al. 2007 [35]	MBSR for FM. Aim: to compare MBSR to an active control including social support, relaxation and stretching exercises	MBSR (5). Home practice assignments	Trained MBSR instructors	Group-based (n = 10–15)	Not reported	8 weeks, 8 2.5-hours, All-day retreat reported	Not reported	Semi-structured individual interviews by instructor before/after intervention on health-related problems and expectations	Of the 58 participants, 6 (10.3%) dropped out (4 from MBSR and 2 from control). All remaining participants completed at least four sessions
Luciano et al. 2014 [36]	Effectiveness of group ACT for FM. Aim: extend findings of Wicksell 2012 with larger sample, longer follow-up and pharmacological control	ACT (7). Home practice assignments	Trained ACT instructors	Group-based (n = 10–15)	Not reported	8 weeks, 8 2.5-hours	Not reported	Video recording of instructors in sessions to insure fidelity. Interview with the participants at baseline	Of 142 participants randomized into 3 groups 20 dropped out of the study. 45 (88%) in GACT, 44 (85%) in RPR, and 47 (89%) in WL completed the study
Parra Delgado et al. 2013 [37]	Effectiveness of MBCT in the treatment of FM. Aim: to examine whether MBCT may reduce the impact of the illness	MBCT (6). Home practice assignments	Trained MBCT instructors	Group-based (n = 17)	Not reported	8 weeks, 8 2.5-hours, All-day retreat not reported	Pain experience acceptance in different mediation practices, awareness of pain-related automatic thought, information on anxiety	Not reported	15 of 17 randomized to intervention group, participated. Drop-out reasons not explained. Ten attended six or more sessions (one attended four, sessions, four five, five six, three seven and two eight sessions. Controls: treatment-as-usual (n = 16), no drop-out
Schmidt et al. 2011 [38]	MBSR on FM. Aim: to include control group to replicate and extend earlier trials lacking randomization or control group	MBSR (5). Home practice assignments	Trained MBSR instructor	Group-based (n = 12)	University	8 weeks, 8 2.5-hours, All-day retreat reported	Not reported	Semi-structured individual interviews by instructor before/after intervention to help participants formulate realistic individual goals for the intervention	Of 137 participants, 25 (18%) dropped out. Similar attendance rate for both interventions (three-armed RCT)
Septhon et al. 2007 [39]	Evaluate whether MBSR provides advantage over standard treatment for depressive symptoms. Aim: to test the effects of MBSR on depressive symptoms	MBSR (5). Home practice assignments	Trained MBSR instructor	Group-based (n = 10–12)	Not reported	8 weeks, 8 2.5-hours, All-day retreat reported	Not reported	Attendance monitored and absent participants received phone call reminder for subsequent sessions	Of 91 treatment participants, 42 (46%) were considered to have completed MBSR during at least 4 of 8 weekly group sessions. Nine attended 4 sessions (18%)
Simister et al. 2018 [42]	RCT of Online ACT for FM. Aim: to evaluate the efficacy of an online ACT protocol	Online ACT (7). Homework exercises	Online platform with seven modules. Each contained written content, mp3 files and videos developed for each module	Online	Access to computer	Participants had two months to complete the program, encouraged to use approx. one week to complete each module	Online ACT protocol modified after clinical pilot study	Treatment team provided weekly e-mail reminders to complete the program and a reminder to contact a team member if any questions or concerns	All 67 intervention group participants accessed the program during treatment period. 60% practiced exercises from ACT components at least once per day, 80% more than once a week
Wicksell et al. 2012 [40]	ACT for FM Aim: to evaluate the efficacy of ACT for FM	ACT (7)	Trained ACT instructors	Group-based (n = 6)	Not reported	12 weeks, 12 1,5-hours sessions	Not reported	If unable to attend a group session, individual 30-min session summary was provided prior to next session. Video recording of instructors in sessions to assess treatment integrity	3 of 23 participants (13%) in the intervention group dropped out during treatment. One of 17 dropped out in the waitlist group

FM = fibromyalgia, MBSR = mindfulness-based stress reduction, MBCT = mindfulness-based cognitive therapy, RCT = randomized controlled trial, ACT = acceptance and commitment therapy

Five of the nine included studies, Cash et al. [41], Septhon et al. [39], Luciano et al. [36], Wicksell et al. [40] and Simister et al. [42] described strategies for adherence and fidelity. Cash et al. [41] and Septhon et al. [39] monitored attendance and absent participants received a reminder phone call to attend subsequent sessions. Wicksell et al. [40] provided a 30-minute session summary for participants who were unable to attend. Absence from five sessions resulted in discontinuation of the treatment program as well as exclusion from the study. Simister et al. [42] provided participants with weekly e-mail reminders to complete the program along with a request to contact a team member if they had any questions or concerns. Luciano et al. [36] and Wicksell et al. [40] video recorded the instructors’ activities in the sessions to ensure fidelity and to assess treatment integrity. Luciano et al. [36] reviewed each group session to assess if the instructors followed the treatment manual. Schmidt et al. [38] did not monitor fidelity because they were concerned that it might disrupt or influence the intervention.

The adherence, defined as participants completing at least half of the sessions, was relatively high, i.e. above 80%, in all but one intervention. One trial reported 61% adherence [34] and the online intervention reported 100% adherence [42]. There were no differences in adherence between the interventions that reported systematic strategies to maintain or improve fidelity and interventions that did not report such strategies (S4 Table).

### Health effects

**Fig 2:**
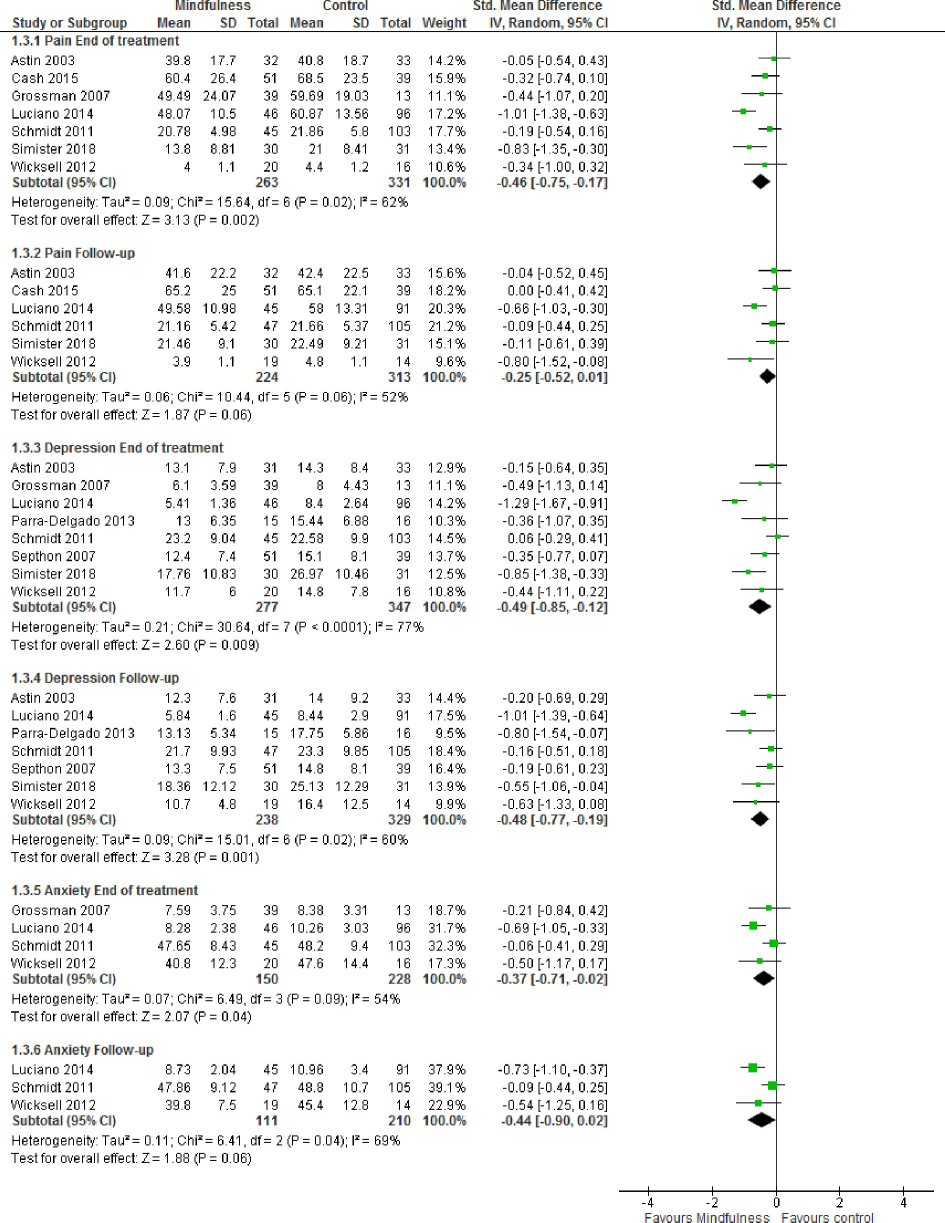
Forest plot for meta-analyses of effects of mindfulness- and acceptance-based interventions. Random-effects meta-analyses of effects of mindfulness- and acceptance-based interventions on pain, depression and anxiety at end of treatment (8-weeks) and follow-up (2–6 months).

**Fig 3:**
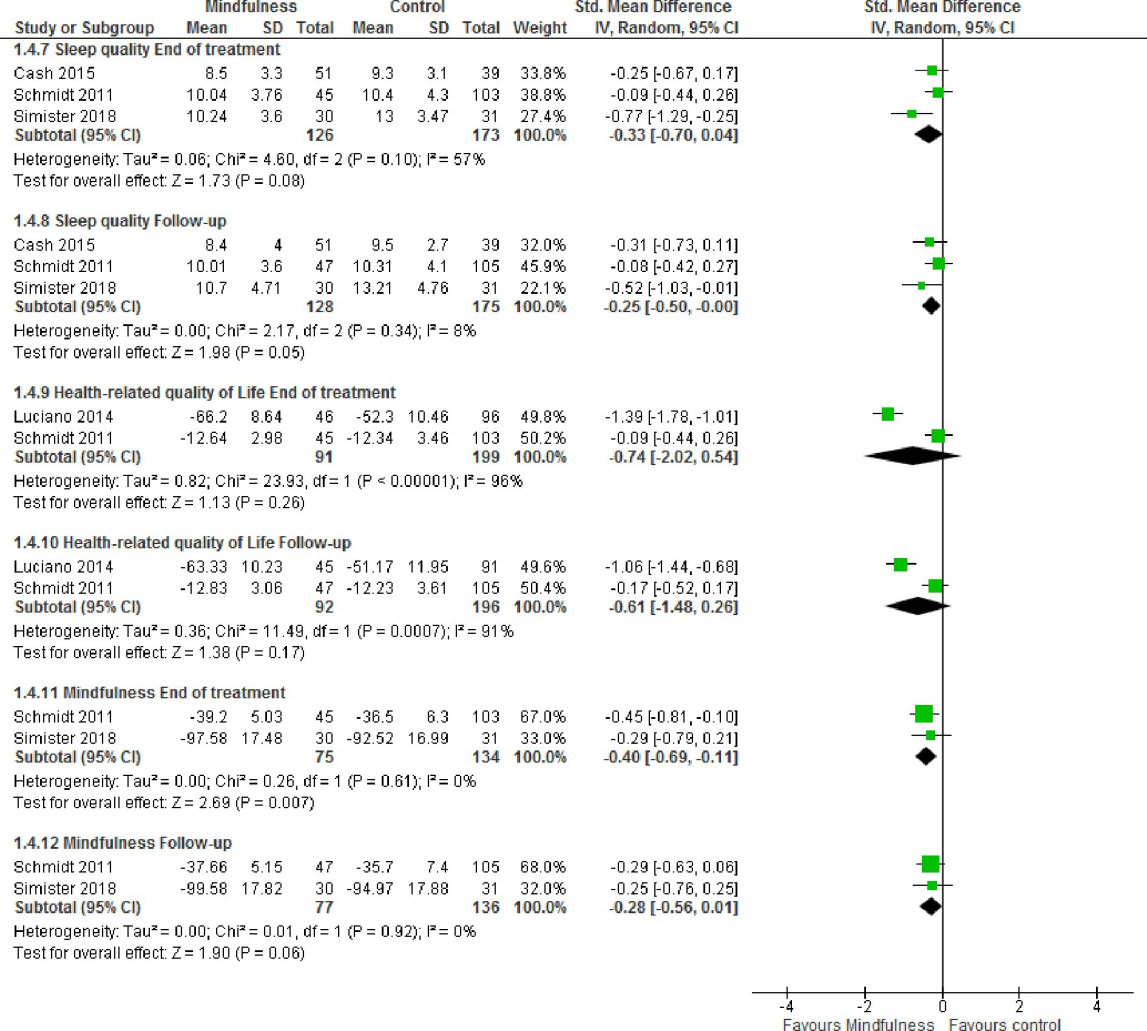
Forest plot for meta-analyses of effects of mindfulness- and acceptance-based interventions. Random-effects meta-analyses of effects of mindfulness- and acceptance-based interventions on sleep quality, health-related quality of life and mindfulness at end of treatment (8-weeks) and follow-up (2–6 months).

The pooled effects were small to moderate in favor of mindfulness- and acceptance-based interventions at the end of treatment for pain (SMD -0.46 [95% CI -0.75, -0.17]), depression (SMD -0.49 [95% CI -0.85, -0.12]), anxiety (SMD -0.37 [95% CI -0.71, -0.02]), sleep quality (SMD -0.33 [-0.70, 0.04]), health-related quality of life (SMD -0.74 [-2.02, 0.54]) and mindfulness (SMD -0.40 [-0.69, -0.11]). At follow-up, all effect sizes decreased except for anxiety that exhibited a small increase in effect size (Fig 2 and Fig 3); (SMD -0.25 [95% CI -0.52, 0.01]), depression (SMD -0.48 [95% CI -0.77, -0.19]), anxiety (SMD -0.44 [95% CI -0.90, 0.02]), sleep quality (SMD -0.25 [-0.50, -0.00]), health-related quality of life (SMD -0.61 [-1.48, 0.26]) and mindfulness (SMD -0.28 [-0.56, 0.01]). Study limitations, inconsistent results and imprecision in the trials (GRADE), resulted in very low to moderate certainty (S5 Table).

## Discussion

The TIDieR-checklist revealed minor differences in content and delivery components among the nine included interventions. The pooled results from nine RCTs totaling 750 patients with FM favored mindfulness- and acceptance-based interventions in all reported outcomes compared to controls. Using the GRADE criteria, the certainty of the evidence was down-graded due to study limitations, inconsistent results and imprecision. These results correspond to another recent systematic review analyzing the effects of CBTs, including ACT and MBCT, on FM [17]. The majority of the included trials applied the ACR 1990 diagnostic criteria which are favorably disposed towards women [27]. The vast majority of the study participants were middle-aged women. The result from this systematic review cannot be generalized to younger women or to men. As the ACR 2010 diagnostic criteria identify many more male patients, future trials should aim to include more men [43].

Using the TIDieR-checklist we found that the included trials provided short descriptions of the interventions and the procedures used (Item 3: Informational materials delivered and Item 4: Procedure used in the intervention) [23]. However, the trial authors referred to the originators of the interventions for further descriptions. All interventions except one were delivered as face-to-face group interventions. Simister et al. [42] delivered an individual online ACT-intervention with therapist support. Online delivery can make interventions more accessible to patients e.g. in rural areas since logistic barriers such as travel or limited access to trained therapists are eliminated [44]. As reported in S8, Simister et al. [42] obtained 100% adherence which might support further development and exploration of online interventions for FM in future research.

Crane et al. [19] have proposed that clarity regarding fidelity to the content and program structure is essential in research on mindfulness- and acceptance-based interventions. Reviewing the TIDieR-checklist Item 12 (How well intervention adherence and fidelity was assessed) we found no differences in actual adherence between trials that reported systematic strategies to improve or maintain fidelity and adherence and the trials that did not report such strategies. All over, actual adherence was relatively high in the included trials. Adherence might be influenced by other aspects that are not explored in this review or not reported in the trials. Since the included treatment interventions often deal with personal emotional issues and require an atmosphere of trust, monitoring fidelity might be disruptive or influence the process [38]. However, we believe that strategies to improve or maintain fidelity and adherence are of importance and might contribute to ensure the core intentions of the interventions in future research [19].

Fatigue and work ability were predefined outcomes in our review. Importantly; none of the included trials measured work ability, even though previous research has demonstrated that FM can have a high impact on work ability [45]. Only one trial [41] measured fatigue and consequently, this outcome was not included in the meta-analysis. Three of the nine included trials, Schmidt et al. [38], Cash et al. [41] and Simister et al. [42], measured sleep quality. Fatigue and sleep disturbances are common in FM [1], and can lower the pain threshold, trigger musculoskeletal pain and increase emotional distress [46]. Future research should include measurements for pain, fatigue, sleep and health-related quality of life to evaluate whether mindfulness- and acceptance-based interventions are more effective for particular outcomes for patients with FM [16, 47].

We did not include the Fibromyalgia Impact Questionnaire (FIQ) which captures the overall impact of FM symptomatology [48]. The reason for excluding FIQ was that it does not allow separate measures of pain, fatigue, depression and anxiety.

A previous systematic review and meta-analysis on mindfulness- and acceptance-based interventions for patients with chronic pain conditions have reported beneficial long-term effects on pain, depression, anxiety and health-related quality of life [15]. The longest follow-up assessment in the trials included in our review was six months. Future studies with longer follow-up periods are needed.

We estimated tau-squared using both the DL and the SJ estimator. The Knapp-Hartung approach (SJ) is known to suit systematic reviews including few studies, particularly when dealing with 5 or fewer trials [49]. The RevMan uses the DL method and is known as the standard estimator [31]. We have chosen to report the results from the meta-analyses computed in RevMan since the results were mainly similar and the small differences had no influence on the conclusion.

To our best knowledge, this review is the first to use the TIDieR-checklist to describe and explore content and delivery components in mindfulness- and acceptance-based interventions. The included trials met fairly rigorous criteria. Some studies were excluded because ACR diagnostic criteria had not been followed or the randomization procedures were not clearly reported. We contacted the authors of these studies and if they did not respond, the trials were excluded.

The TIDieR-checklist may be appropriate to use in conjunction with the CONSORT-checklist [50] when reporting randomized controlled trials. We expected that the TIDieR-checklist would be appropriate for description and exploration of mindfulness- and acceptance-based interventions. MBSR and MBCT are relatively clearly described and manualized interventions and major differences were not revealed in any TIDieR items except for the items describing fidelity and adherence as discussed above. The TIDieR-checklist might be more suitable for exploring more complex interventions that are not described in manuals. Nevertheless, the TIDieR-checklist allows the authors to better describe interventions in sufficient details to make replication possible [23].

Adopting the optimal version of mindfulness- and acceptance-based interventions might be difficult due to the heterogeneity between the included trials [22]. Some of the pooled effects were also based on a small number of trials. Users of mindfulness- and acceptance-based interventions should consider the effects against risk of bias, adherence, and fidelity in the included trials (Tables 1, 2) [20]. Future trials should investigate whether strategies to improve adherence and fidelity of mindfulness- and acceptance-based interventions can improve health outcomes.

## Conclusions

Overall, the mindfulness- and acceptance-based interventions included in this review were associated with small to moderate uncertain effects on pain, depression, anxiety, sleep quality, health-related quality of life and mindfulness for female patients with FM compared to controls. In our review we found no association between the mindfulness- and acceptance-based interventions that reported strategies to improve adherence and fidelity and trials that did not report such strategies. Future trials should monitor adherence and fidelity to explore this association more extensively. There was heterogeneity between trials, and down-grading in GRADE resulted in very low, low and moderate certainty of evidence. Only limited conclusions can therefore be drawn. The TIDieR-checklist is a useful supplement that can improve the reporting of RCTs but might be less informative for manualized interventions.

## Supporting information

S1 Table TIDieR-checklist. (PDF)

S2 Table Data extraction form created for the review. (PDF)

S3 Table Excluded studies with reasons. (XLSX)

S4 Table Item 11 and 12 in TIDieR-checklist specified. (PDF)

S5 Table GRADE evidence profile. (PDF)

S6 Table PRISMA-checklist. (PDF)

S1 Text Search strategy for MEDLINE. (PDF)

S1 Fig Risk of bias summary. The review authors' judgements of the risk of bias of each included study.
(PDF)
