# Supplementary material for: Mindfulness- and acceptance-based interventions for patients with fibromyalgia – A systematic review and meta-analyses
Source: PLoS One. 2019 Sep 3;14(9):e0221897. doi: 10.1371/journal.pone.0221897 (PMC6719827; doi:10.1371/journal.pone.0221897)
Supplement: S1 Table — (PDF) [file pone.0221897.s001.pdf]

TIDieR-checklist

Author Year	Brief name	Recipient	Why	What (materials)	What (procedures)	Who provided	How	Where	When and how much	Tailoring	Modification of intervention throughout trial	Strategies to improve or maintain intervention fidelity	Extent of intervention fidelity
