# Supplementary material for: Mindfulness- and acceptance-based interventions for patients with fibromyalgia – A systematic review and meta-analyses
Source: PLoS One. 2019 Sep 3;14(9):e0221897. doi: 10.1371/journal.pone.0221897 (PMC6719827; doi:10.1371/journal.pone.0221897)
Supplement: S2 Table — (PDF) [file pone.0221897.s002.pdf]

Data extraction form (trial)

(Title)

1. General Information

**Person extracting data (name)**	
**Date form completed**	
**Report title** *(title of paper/ abstract/ report that data are extracted from)*	
**Study country**	

2. Population and setting

**Recruitment details**	Number of people screened:<br>Number of people eligible:<br>Number of people recruited:<br>Number of people randomized:
**Notes:**	

3. Methods

	Descriptions as stated in report/paper	Location in text *(pg & ¶/fig/table)*
**Aim of study**		
**Design** *(e.g. parallel, cluster)*		
**Control group** (What did the control group receive?)	**Wait-list** ☐ **no intervention** ☐ **treatment as usual** ☐<br>**Other active treatment:**<br>**Describe:**	
**Notes:**		

4. Risk of Bias assessment

See [Chapter 8] of the Cochrane Handbook and ***The Cochrane collaboration tool for assessing risk of bias; criteria for judging risk of bias in the 'Risk of bias' assessment tool***

**Domain**	**Risk of bias**<br><br>Low risk	 <br>High risk	 <br>Unclear	**Support for judgement (describe)**	**Location in text** *(pg & ¶/fig/table)*
**Random sequence generation** *(selection bias)*	☐	☐	☐		
**Allocation concealment** *(selection bias)*	☐	☐	☐		
**Blinding of participants and personnel** *(performance bias)*	☐	☐	☐	**Outcome group: All/**	
**Blinding of outcome assessment: self-reported outcomes** *(detection bias)*	☐	☐	☐	**Outcome group: All/**	
**Blinding of outcome assessment: objective outcomes** *(detection bias)*	☐	☐	☐	**Outcome group: All/**	
**Incomplete outcome data** *(attrition bias)*	☐	☐	☐		
**Selective outcome reporting?** *(reporting bias)*	☐	☐	☐		
**Other bias**	☐	☐	☐		
**Notes:**					

5. Participants

Provide overall data and, if available, comparative data for each intervention or comparison group.

	**Setting;**			**Location in text** (pg & ¶/fig/table)
**Specific intervention**				
**Number of participants**	**Total:**	**Intervention:**	**Comparison:**	
**% Female**	**Total:**	**Intervention:**	**Comparison**	
**Age**	**Total:**	**Intervention:**	**Comparison:**	

**Diagnose criteria**				
**Sample size**		**Intervention:**	**Comparison**	
**Comments from the authors**				
**Notes:**				

6. Outcomes / results **(In Revman)**

Copy and paste table for each outcome

Primary time point

	Outcome	Result	Location in text *(pg & ¶/fig/table)*
Post-intervention	Pain		
	Fatigue		
	Sleep quality		
	Psychological stress		
	Depression		
	Anxiety		
	Mindfulness		
	Quality of Life		
	Work participation ability		

	Description as stated in report/paper	Location in text *(pg & ¶/fig/table)*
Key conclusions of study authors		
Notes:		

**Adverse Events**	

Exclusion after data extraction

Reasons for exclusion: (study design? participants? interventions/ outcomes? attrition? bias?)

Dates:

Date entered into RevMan and by whom?

Date checked and by whom?
