# Supplementary material for: Mindfulness- and acceptance-based interventions for patients with fibromyalgia – A systematic review and meta-analyses
Source: PLoS One. 2019 Sep 3;14(9):e0221897. doi: 10.1371/journal.pone.0221897 (PMC6719827; doi:10.1371/journal.pone.0221897)
Supplement: S4 Table — (PDF) [file pone.0221897.s004.pdf]

Table over item 11 and 12 in TIDieR-checklist specified

Authors	Strategies to improve or maintain intervention adherence and fidelity	Extent of intervention adherence	Effect size, follow-up
Luciano et al. (36) (ACT)	Instructors were video recorded, participants were interviewed before intervention	n=45: 88% completed the intervention	Pain: -0.66, depression: -1.01, anxiety: -0.73, health-related quality of life: -1.06
Wicksell et al. (40) (ACT)	Instructors were video recorded. If unable to attend a session, 30-minutes summary were provided	n=19: 87% completed the intervention	Pain: -0.80, depression: -0.63, anxiety: -0.54
Simister et al. (42) (Online ACT)	E-mail reminders to participants	n=30: 100% completed the intervention	Pain: -0.11, depression: -0.55, sleep quality: -0.52, mindfulness: -0.25
Cash et al. (41) (MBSR)	Attendance monitored and absence tracked	n=51: 82% completed at least 4 of 8 sessions	Pain: 0.00, sleep quality: -0.31
Septhon et al. (39) (MBSR)	Attendance monitored and absence tracked	n=51: 82% completed at least 4 of 8 sessions	Depression: -0.19
Astin et al. (34) (MBSR)	No strategies reported	n=31: 61% completed the intervention	Pain: -0.04, depression: -0.20
Grossmann et al. (35) (MBSR)	No strategies reported	n=39: 88% completed at least 4 of 8 sessions	*Pain: -0.01, depression: -0.49, anxiety: -0.21,
Schmidt et al. (38) (MBSR)	No strategies reported	n=45: 89% completed at least 4 of 8 sessions	Pain: -0.09, depression: -0.16, anxiety: -0.09, sleep quality: -0.08, health-related quality of life: -0.17, mindfulness: -0.29
Parra Delgado et al. (37) (MBCT)	No strategies reported	n=15: 88% completed the intervention	Depression: -0.80

*End of treatment data.
