# Supplementary material for: Mindfulness- and acceptance-based interventions for patients with fibromyalgia – A systematic review and meta-analyses
Source: PLoS One. 2019 Sep 3;14(9):e0221897. doi: 10.1371/journal.pone.0221897 (PMC6719827; doi:10.1371/journal.pone.0221897)
Supplement: S5 Table — (PDF) [file pone.0221897.s005.pdf]

GRADE evidence profile

Certainty assessment							№ of patients		Effect		Certainty
№ of studies	Study design	Risk of bias	Inconsistency	Indirectness	Imprecision	Other considerations	Mindfulness	control	Relative (95% CI)	Absolute (95% CI)	
**Pain End of treatment**											
7	randomised trials	serious [a]	serious [b]	not serious	not serious	none	263	331	-	SMD **0.46 SD lower** (0.75 lower to 0.17 lower)	⊕⊕◯◯ LOW
**Pain Follow-up**											
6	randomised trials	serious [c]	not serious	not serious	serious [d]	none	224	313	-	SMD **0.25 SD lower** (0.52 lower to 0.01 higher)	⊕⊕◯◯ LOW
**Depression End of treatment**											
8	randomised trials	serious [a]	serious [b]	not serious	not serious	none	277	347	-	SMD **0.49 SD lower** (0.85 lower to 0.12 lower)	⊕⊕◯◯ LOW
**Depression Follow-up**											
7	randomised trials	serious [c]	serious [b]	not serious	not serious	none	238	329	-	SMD **0.48 SD lower** (0.77 lower to 0.19 lower)	⊕⊕◯◯ LOW
**Anxiety End of treatment**											
4	randomised trials	serious [a]	not serious	not serious	not serious	none	150	228	-	SMD **0.37 SD lower** (0.71 lower to 0.02 lower)	⊕⊕⊕◯ MODERATE
**Anxiety Follow-up**											
3	randomised trials	serious [e]	serious [b]	not serious	serious [d]	none	111	210	-	SMD **0.44 SD lower** (0.9 lower to 0.02 higher)	⊕◯◯◯ VERY LOW

**Sleep quality End of treatment**											
3	randomised trials	serious [e]	not serious	not serious	serious [d]	none	126	173	-	SMD **0.33 SD lower** (0.7 lower to 0.04 higher)	⊕⊕◯◯ LOW
**Sleep quality Follow-up**											
3	randomised trials	serious [e]	not serious	not serious	not serious	none	128	175	-	SMD **0.25 SD lower** (0.5 lower to 0)	⊕⊕⊕◯ MODERATE
**Health-related quality of Life End of treatment**											
2	randomised trials	serious [e]	serious [b]	not serious	serious [d]	none	92	152	-	SMD **0.62 SD lower** (1.52 lower to 0.28 higher)	⊕◯◯◯ VERY LOW
**Health-related quality of Life Follow-up**											
2	randomised trials	serious [e]	serious [b]	not serious	serious [d]	none	92	152	-	SMD **0.62 SD lower** (1.52 lower to 0.28 higher)	⊕◯◯◯ VERY LOW
**Mindfulness End of treatment**											
2	randomised trials	serious [e]	not serious	not serious	not serious	none	75	134	-	SMD **0.4 SD lower** (0.69 lower to 0.11 lower)	⊕⊕⊕◯ MODERATE
**Mindfulness Follow-up**											
2	randomised trials	serious [e]	not serious	not serious	serious [d]	none	77	136	-	SMD **0.28 SD lower** (0.56 lower to 0.01 higher)	⊕⊕◯◯ LOW
**Fatigue - not measured**											
									-	**0** (0 to 0)	-
**Workability - not measured**											
									-	**0** (0 to 0)	-

CI: Confidence interval; **SMD:** Standardised mean difference

Explanations

a. The Risk of Bias show High Risk on attrition bias, selection bias and performance bias. In addition there are some uncertainty and unclear risk.

b. The p-value is low and the I-square is high.

c. The Risk of Bias show High Risk on attrition bias and performance bias. In addition there are some uncertainty and unclear risk.

d. The confidence interval goes from a large effect in favor of mindfulness to a small effect in favor of control.

e. The Risk of Bias show High Risk on performance bias. In addition there are some uncertainty and unclear risk.
