# Supplementary material for: Mindfulness- and acceptance-based interventions for patients with fibromyalgia – A systematic review and meta-analyses
Source: PLoS One. 2019 Sep 3;14(9):e0221897. doi: 10.1371/journal.pone.0221897 (PMC6719827; doi:10.1371/journal.pone.0221897)
Supplement: S1 Text — (PDF) [file pone.0221897.s007.pdf]

MEDLINE

Search Strategy:

----

1 Fibromyalgia/ (8417)

2 Chronic Pain/ (10259)

3 fibromyalg*.tw,kf. (9869)

4 (chronic adj1 widespread adj1 pain).tw,kf. (803)

5 or/1-4 (21108)

6 Mindfulness/ (1728)

7 "Acceptance and Commitment Therapy"/ (230)

8 mindfulness*.tw,kf. (4958)

9 (acceptance and commitment therap*).tw,kf. (648)

10 ((awareness adj6 attention) and (medit* or vipassana)).tw,kf. (75)

11 or/6-10 (5692)

12 5 and 11 (235)

13 randomized controlled trial.pt. (505234)

14 controlled clinical trial.pt. (100418)

15 (randomized or placebo or randomly or trial or groups).ab. (2593502)

16 or/13-15 (2740891)

17 12 and 16 (111)

18 limit 17 to yr="1990 -Current" (111)
