# Supplementary material for: Mindfulness- and acceptance-based interventions for patients with fibromyalgia – A systematic review and meta-analyses
Source: PLoS One. 2019 Sep 3;14(9):e0221897. doi: 10.1371/journal.pone.0221897 (PMC6719827; doi:10.1371/journal.pone.0221897)
Supplement: S1 Fig — The review authors' judgements of the risk of bias of each included study. (PDF) [file pone.0221897.s008.pdf]

Risk of bias summery

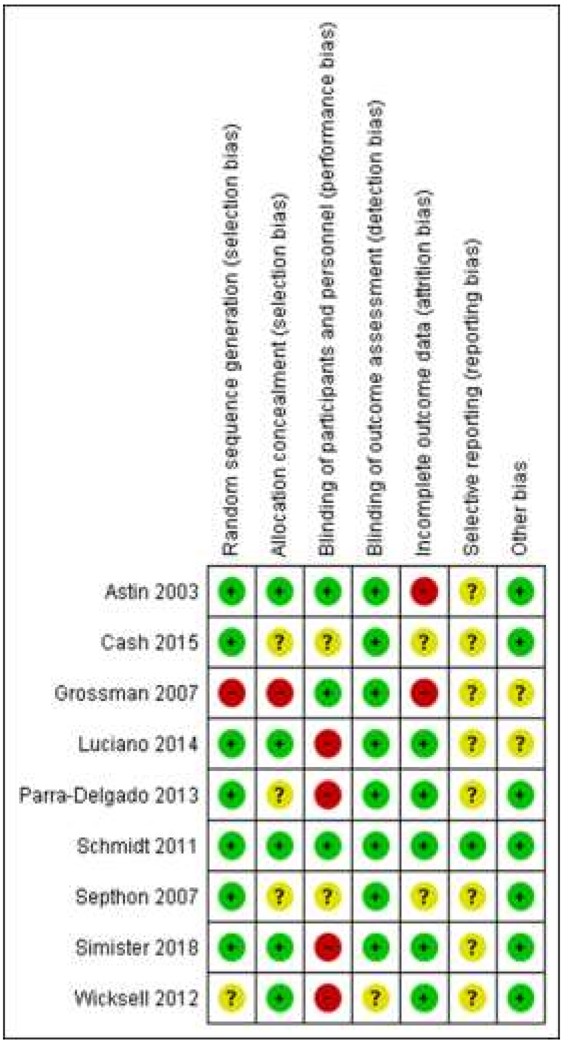

Reasons for high risk of bias

Astin 2003 et al. (34): Incomplete outcomes; 26 % never attended a class. Of 128 randomized into 2 groups, 50 (39%) dropped out from the study prior to 'end of treatment', 61 (48%) dropped out by week 16, and 63 (49%) failed to complete 24 week assessment.

Grossman 2007 et al. (35): Random sequence generation; quote p. 228: 'A quasi-random allocation to treatments was based upon alternation of small groups of patients according to time of enrollment'. Allocation concealment; Alternation of small groups of patients according to time of enrollment. The first 31 patients who enrolled comprised the initial two MBSR groups; the next consecutive 15 patients formed the control group, which was then followed with subsequent MBSR groups. Incomplete outcomes; 26 of 58 participants in follow-up.

Luciano 2014 et al. (36): Blinding of participants and personnel; Not blinded.

Parra-Delgado 2013 et al. (37): Blinding of participants and personnel; Participants were probably aware of assignment.

Simister 2018 et al. (42): Blinding of participants and personnel; quote p. 743: 'a registered physiotherapist...remained blind to participants' assigned condition for the duration of the study'. Participants were probably aware of assignment

Wicksell 2012 et al. (40): Blinding of participants and personnel; Wait-list control.
